# Is the Insect World Overcoming the Efficacy of *Bacillus thuringiensis*?

**DOI:** 10.3390/toxins9010039

**Published:** 2017-01-18

**Authors:** Cecilia Peralta, Leopoldo Palma

**Affiliations:** 1 Centro de Investigaciones y Transferencia de Villa María (CITVM-CONICET), Universidad Nacional de Villa María, 5900 Villa María, Córdoba, Argentina; ceci036@gmail.com; 2 Consejo Nacional de Investigaciones Científicas y Técnicas, C1 425FQB Ciudad Autónoma de Buenos Aires, Argentina

**Keywords:** *Bacillus thuringiensis*, insecticidal toxins, biological control, insect pests, field-evolved resistance

## Abstract

The use of chemical pesticides revolutionized agriculture with the introduction of DDT (Dichlorodiphenyltrichloroethane) as the first modern chemical insecticide. However, the effectiveness of DDT and other synthetic pesticides, together with their low cost and ease of use, have led to the generation of undesirable side effects, such as pollution of water and food sources, harm to non-target organisms and the generation of insect resistance. The alternative comes from biological control agents, which have taken an expanding share in the pesticide market over the last decades mainly promoted by the necessity to move towards more sustainable agriculture. Among such biological control agents, the bacterium *Bacillus thuringiensis* (Bt) and its insecticidal toxins have been the most studied and commercially used biological control agents over the last 40 years. However, some insect pests have acquired field-evolved resistance to the most commonly used Bt-based pesticides, threatening their efficacy, which necessitates the immediate search for novel strains and toxins exhibiting different modes of action and specificities in order to perpetuate the insecticidal potential of this bacterium.

The use of chemical pesticides began in the 1940s with the introduction of DDT (Dichlorodiphenyltrichloroethane) as the first modern chemical insecticide, which played a crucial role in crop protection and controlling human-disease vectors [1]. However, its effectiveness, together with its low cost have favoured the indiscriminate use of DDT and other synthetic insecticides with the generation of undesirable side effects, such as the contamination of water and food sources, harm to non-target insects (e.g., natural predators, pollinators, etc.) and the generation of insect resistance to these pesticides. After some time, it was realized that by reducing wide-spectrum pesticide levels in the field, some beneficial insects such as natural enemies of pests, were allowed to survive, thereby contributing to a combined method for the control of undesirable insect pests in agriculture. This ‘mixed’ method of control, conceived around the 1950s, was the first concept of integrated pest management (IPM) [2]. In addition, any living organism is susceptible to diseases caused by the action of pathogenic microorganisms (e.g., viruses, bacteria, fungi, etc.) therefore, entomopathogenic microorganisms can be also used along with synthetic insecticides for the control of insect pests, human-disease vectors and other annoying insects. The insect-pathogenic microorganisms are highly specific to their hosts and many can be mass-produced (e.g., baculoviruses, entomopathogenic bacteria, entomopathogenic nematodes, etc.). The biologically active biomass constitutes the active ingredient of these environmentally friendly formulations, which are commonly known as microbial insecticides or bio-insecticides. In this context, Gram-positive bacteria have received considerable attention during the last decades because of their ability to produce a number of insecticidal toxins suitable for the control of both crop pests and human-disease vectors (principally mosquitoes). Since the discovery of *Bacillus thuringiensis* (Bt) a century ago [3], other entomopathogenic Gram-positive bacteria were also identified: *Lysinibacillus sphaericus, Bacillus cereus, Paenibacillus popilliae* and *Clostridium bifermentans*. However, Bt has been the most studied bacterium and is the first one to have been marketed since 1938 [4].

Bt is a Gram-positive, spore-forming bacterium that synthesizes several insecticidal proteins active against a wide range of insect pests and has demonstrated its potential and safety as a biocontrol agent for several decades [5]. The insecticidal proteins synthesized by this bacterium include both crystal proteins, commonly known as delta-endotoxins (including Cry and Cyt toxins), and the vegetative insecticidal proteins (Vip) including Vip1, Vip2 and Vip3 [6,7,8]. Vip1 and vip2 proteins constitute binary toxins mainly active against Coleoptera whereas Vip3 proteins are active against Lepidoptera [9]. Each single Cry toxin is very specific, exhibiting a narrow host range while in general they are active against a wide range of invertebrates [8]. The mode of action (MOA), best studied in Lepidoptera and for Cry toxins, involves crystal ingestion and solubilization in the alkaline insect midgut, the proteolytic activation of the protoxin and binding of the toxin to specific receptors located on the surface of midgut epithelial cells, leading to pore formation and causing cell lysis [10] (Figure 1). The Vip3 MOA, although remaining poorly understood, resembles the MOA of Cry toxins but differs in the cell receptor used for toxin binding, opening the possibility of using combinations of both toxins for increased toxin activity (synergism), broadening host spectrum and managing insect resistance [11,12]. Cry and Cyt proteins are produced during sporulation as parasporal inclusions and are typically found in this bacterium [7]. This feature has allowed the production of sprayable Bt-based biopesticides wherein the protein crystals constitute the active ingredient of the bioinsecticide. Secretable proteins are produced during the vegetative growth and are later secreted (and diluted) into the culture medium [13]. This behaviour has prevented their commercial production as formulated biopesticides relegating their use to the construction of transgenic crops exhibiting resistance to susceptible insect pests (e.g., Vip cotton, etc.) [14]. To date, more than 70 different classes of Cry proteins have been described, from Cry1 to Cry74 [5]. The extraordinary diversity of insecticidal genes harboured by this bacterium is likely to have been produced as the result of selective evolutionary pressures exerted by susceptible insects [6,15,16], leading to the enlargement on target ranges or by generating new specificities. However, the susceptible insects must also be suffering selective pressures as demonstrated by the rapid evolution of insect resistance to some of the most used insecticidal strains and toxins (e.g., Cry1A and Cry2A) [17,18,19].

Sprays containing combinations of Cry1A and Cry2A toxins have been used in agriculture for several years and despite the fact that there are a few reports concerning insect resistance over Bt-based formulations, field-evolved resistance has occurred promoted by the selective pressure exerted over some insect populations (e.g., the lepidopteran *Trichoplusia ni* and DiPel formulation) [19]. Nevertheless, this phenomenon more frequently affects the most used Bt crops in agriculture, especially those from the first generation, which express only one Cry protein. In order to address field-evolved resistance problems, second generation (2^nd^ g) Bt crops were designed and produced. These 2^nd^ g Bt crops are capable of expressing two different types of Bt toxins (e.g., Cry1 and Cry2 or Cry1 and Vip3) that kill the same insect pests (e.g., some lepidopterans) [20]. Such combinations should help to prevent the development of insect resistance and also broaden the insect target range of a given Bt crop. Nevertheless, taking into account the demonstrated potential of the insects to adapt and therefore, tolerate chemical and biological insecticides, it may be a matter of time before the generation of resistance to 2^nd^ g Bt plants. In fact, some reports indicate the reduced efficacy of 2^nd^ g Bt cotton and corn harbouring Cry1Ac + Cry2Ab and Cry1A.105 + Cry2Ab against *Helicoverpa zea* and *Spodoptera frugiperda*, respectively [21,22]. Such resistance events are reducing Bt crops efficacy, requiring novel strategies that could anticipate the evolutionary responses of insect pests [23]. In this context, PCR screening programs have successfully identified several Bt strains and potential insecticidal genes, that after toxicity tests, exhibited activity against a wide range of invertebrates [5,24]. However, PCR-based strategies are time consuming and limited, since for instance, a pair of degenerated PCR primers will have a narrow range for only one kind of gene target (e.g., *cry*, *cyt* or *vip*) [5]. Nowadays, with the availability of Next-Generation Sequencing technologies, searching programs for novel toxin genes have become faster, cheaper and more efficient, providing also additional genomic information and opening the possibility of finding as yet unknown insect-active toxins suitable for the production of novel biological pesticide formulations or the construction of transgenic plants with effective resistance to insect pests.

Novel insecticidal proteins with different modes of action are interesting alternatives to managing insect resistance since they can be useful to overcome the three main causes inducing insect resistance [17] (1) Alterations at the proteolytic processing site needed for pro-toxin activation: changes in midgut proteases can completely abolish or at least reduce toxin activity. The availability of other toxins as protease ‘substrates’ can be successfully used to overcome this problem; (2) The modification of the toxin-binding site (cell receptor): this source of insect resistance happens when modifications into the amino acid sequences of the receptors are produced (e.g., by non-synonymous substitutions that lead to an amino acid change), which can dramatically diminish or completely abolish toxin binding. Thus, the availability of additional or alternative toxins, with different binding sites, can be successfully used to solve these kinds of resistance problems by enhancing toxin activity and broadening host-spectrum if two or more toxins are properly combined; (3) By other less known mechanisms such as a better capability of the insect for the replacement or the repair of damaged midgut cells. The combination of two different toxins can effectively reduce this problem by delaying or abolishing midgut repair.

In addition and beyond toxin proteins, genomic information can also provide valuable information about additional accessory proteins (e.g., P20 and Bel enhancing homologs). The chaperone P20 has been already described to improve or to enhance the activity of some Bt toxins, e.g., Cry11A against third instar larvae of *Aedes aegypti* [25]. Moreover, it also showed interesting capabilities of enhancing expression and crystallization of Cry1Ac toxin [26]. The Bt Bel enhancin protein is a metalloprotease enzyme, which is capable of producing a peritrophic membrane disruption enhancing the activity of Cry1Ac toxin against *Helicoverpa armigera* [27]. In order to prevent or to reduce insect resistance to a given toxin, the P20 accessory proteins and Bel enhacin homologs are potential alternatives to be included in novel Bt-based formulations. Also, their coding genes can be successfully included in novel Bt crops with enhanced resistance to insects since a viral enhancin gene from a baculovirus protects tobacco from the action of *Trichoplusia ni* [28]. In addition, such approaches will be better exploited by addressing the current lack of knowledge concerning the structure and mode of action of these invertebrate-active toxins, which are the indispensable ingredients of these biological control systems [29].

Finally, the insecticidal potential of novel Bt crops could be significantly enhanced by their combination with other insecticidal transgenes coming from Gram-negative entomopathogenic bacteria such as those species from the *Photorhabdus* and *Xenorhabdus* genera (e.g., *Photorhabdus luminescens*). *Photorhabdus* and *Xenorhabdus* bacteria live in a perfect symbiosis with entomopathogenic nematodes from *Heterorhabditis* and *Steneinerma* genera, respectively, and produce several highly toxic proteins against insects [30]. These insecticidal toxins lead to a rapid killing of some insect pests and should be taken into account as potential next-generation transgenes for the control of pests in agriculture. 

Since the discovery of DDT in the 1940s, a number of other chemical pesticides were also produced and used in agriculture, causing the pollution of the agri-ecosystems, affecting non-target insects (e.g., natural enemies of pests and pollinators) and with undesirable side effects to other organisms and humans. The alternative to chemical pesticides came up from different biological control agents capable of causing diseases and killing insect pests in modern agriculture. To date, Bt has been the most studied and used bacterial biological control agent, however, several pest species have acquired field resistance to the most used Bt toxins, and more severely, to those included into transgenic crops. This phenomenon calls for immediate action in order to overcome insect resistance problems and to maintain the insecticidal potential of this bacterium.

## Figures and Tables

**Figure 1:**
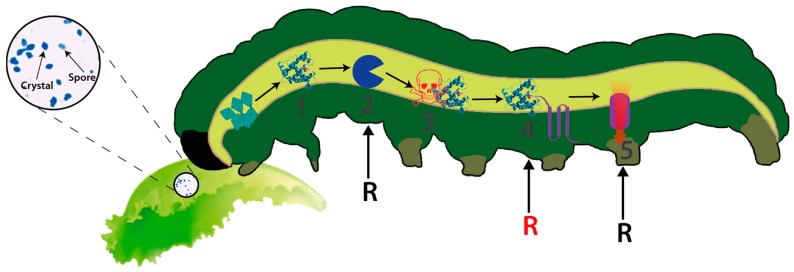
Schematic view representing the MOA of Cry toxins from Bt. (1) Crystal ingestion, solubilisation and free soluble protoxin in the gut; (2) Pro-toxin activation by proteolysis and (3) Free activated toxin on the gut; (4) Toxin binding to cell receptors and (5) Pore formation, osmotic disequilibrium and cell lysis. Black and red R letters indicate the most susceptible steps for the generation of insect resistance in the laboratory (steps 2, 4 and 5) whereas field-evolved resistance generally compromises the binding of the toxin to its receptors (step 4, red R letter).
